# A systematic search and qualitative review of reporting bias of lifestyle interventions in randomized controlled trials of diabetes prevention and management

**DOI:** 10.1186/s12937-018-0390-6

**Published:** 2018-09-07

**Authors:** Natalie D. Riediger, Andrea E. Bombak, Adriana Mudryj, Jackson Bensley, Samuel Ankomah

**Affiliations:** 1 Department of Food and Human Nutritional Sciences, Faculty of Agricultural and Food Sciences, University of Manitoba, 407 Human Ecology Building, Winnipeg, MB R3T 2N2 Canada; 2 Department of Community Health Sciences, Rady Faculty of Health Sciences, University of Manitoba, Winnipeg, MB Canada; 3 Department of Sociology, Faculty of Arts, University of New Brunswick, Fredericton, NB Canada; 4 School of Health Sciences, Community Health Division, Central Michigan University, Mt Pleasant, MI USA

**Keywords:** Diabetes, Lifestyle, Randomized controlled trials, Spin reporting, Reporting bias, Participant adherence, Patient-oriented research

## Abstract

**Background:**

Scholars have documented presumptions regarding the relationships between diet, exercise, weight, and type 2 diabetes. However, it is unclear to what extent researchers contribute to these presumptions, and how often these relationships are thoroughly delineated within the context of randomized controlled trials (RCTs). Thus, the aim was to conduct a systematic search and qualitative, thematic analysis of RCTs focusing on lifestyle interventions for diabetes prevention or management, to examine how researchers discuss body weight in 1) the rationale and design of their RCTs; and 2) their presentation and interpretation of their findings.

**Methods:**

We completed an electronic search for records published between 2007 and November 2016. Selection criteria included: RCTs with a follow-up period of ≥12 months; adult participants with type 2 diabetes/pre-diabetes; lifestyle interventions classified as dietary, exercise, and/or behavioural; primary outcomes of incident diabetes and complications, mortality, cardiovascular disease, and quality of life; and secondary outcomes of glycemic control and blood pressure. Nineteen articles were identified for inclusion and subject to thematic content analysis.

**Results:**

Obesity and weight loss figured prominently in the rationale and outcomes of the majority of the articles, despite intentional exclusion of “weight loss” and “obesity” as search terms. There was ambiguity over whether weight loss was classified as inclusive to the intervention, an outcome, or a measure of adherence. Results revealed that authors frequently engaged in “spin reporting” by pooling data from intervention and control groups to test the relationship between weight lost and outcomes and in their presentation of results.

**Conclusions:**

Researchers need to be aware of their biases and assumptions regarding body weight in designing, analyzing, and interpreting lifestyle interventions for diabetes prevention and management.

## Background

The global prevalence of type 2 diabetes is growing, with 642 million adults estimated to have diabetes in 2014 [1]. Lifestyle changes, specifically, diet and activity, are understood to be important in primary and secondary prevention of diabetes complications. Lifestyle recommendations are based mostly on observational epidemiological studies in which causality cannot be inferred. Randomized controlled trials (RCTs) are viewed as one of the highest forms of evidence [2], and intervention research following this method is thought to potentially alleviate the methodological concerns of observational data. The Diabetes Prevention Program (DPP), one of the earliest RCTs of lifestyle for diabetes prevention, helped to establish that lifestyle changes could delay the onset of type 2 diabetes by 4 years in participants with overweight or obesity and impaired glucose tolerance [3]. Weight loss was determined to be the most important contributor to the lifestyle intervention effect [4]. Subsequently, diabetes prevention programs have been translated into multiple settings, often with a focus on participants with overweight and obesity.

Obesity is a risk factor for type 2 diabetes [5], though type 2 diabetes does affect individuals of all sizes. Despite the potential for individuals of any size to develop diabetes, diabetes is often conflated with obesity; both conditions are frequently depicted as caused by similar dietary and exercise-related choices [6]. This conflation has important implications for how diabetes is presented and treated in research, practice, and policy. Scholars have begun to call attention to the myriad of myths and presumptions, the imprecision of measures, lowered validity claims, and the non-specified etiological mechanisms in obesity research [7–9]. Obesity research may be particularly susceptible to these elisions because obesity is also a stigmatized and morally-laden condition. Obesity is often presented in reductionist terms as a matter of personal responsibility caused by a simplified energy imbalance model [10, 11].

While some view more RCTs as one way of rectifying issues concerning validity, objectivity, and precision [9], even well-designed, carefully interpreted, and valuable RCTs do not exist in a vacuum; many limitations of RCTs have been reported in the literature [12–16]. Systematic reviews and corresponding quantitative meta-analysis of RCTs, the apex of the evidence hierarchy, are designed to minimize and also detect bias, such as selection bias, performance bias, detection bias, attrition bias, and reporting bias [17]. Reporting bias can be further categorized as publication bias, time lag bias, multiple publication bias, location bias, citation bias, language bias, and outcome reporting bias [17]. Unfortunately, quantitative methods to detect various biases are not well-developed. While the Cochrane Handbook has described areas for qualitative analysis in systematic reviews, this is limited to the systematic review of qualitative research in the same content area of the corresponding meta-analysis of RCTs. Qualitative methods may be useful for systematically analysing bias, particularly reporting bias that is potentially influenced by the social stigmatization of obesity and reductionist approaches to obesity research.

What we detected in the present analysis has previously been labelled “spin reporting” by Boutron and colleagues [16]. Spin reporting is defined as “reporting that can distort the interpretation of results and mislead readers”, usually to present the experimental treatment as effective. This concept encompasses a broad range of reporting issues, including, but not limited to, focussing on statistically significant results, interpreting non-significant findings as demonstrating comparable effectiveness, or asserting beneficial treatment effects despite non-significant findings [16]. Spin reporting may in turn contribute to the imprecision and assumptions that underlay “diabesity research” more generally. Thus, the aim was to conduct a systematic search for RCTs focusing on lifestyle interventions for diabetes prevention or management, while purposefully excluding body weight-related terms in our search strategy, to uncover the extent to which measures of body weight are conflated with diabetes. Using a qualitative, thematic analysis, we then sought to examine how researchers discussed body weight in 1) the rationale and design of their RCTs; and 2) their presentation and interpretation of their findings.

## Methods

### Design and search strategy

We conducted a systematic search for peer reviewed journal articles using the following electronic databases: CINAHL, EMBASE, PsychInfo, PubMed, and Web of Science. We searched for articles published in English between January, 2007 and November, 2016. These dates were selected to represent current research in the area. Study selection keywords included those related to type II diabetes mellitus (such as “diabetes mellitus, type 2”) as well as keywords related to lifestyle interventions, used in the prevention or management of type 2 diabetes (including, but not limited to terms such as “exercise”, “nutrition”, “diet therapy” etc.). Search term selection was modelled after a Cochrane Review [18], though the terms, “obesity”, “body weight”, “body mass index”, and “weight loss” were purposefully not included in the search. A complete list of search terms can be found in Appendix 1. We also completed a hand search following the systematic search. This systematic review is not registered.

### Inclusions/exclusion criteria

Selection criteria included: RCTs with a follow-up period of at least 12 months. Participants were persons ≥18 years of age with type 2 diabetes or pre-diabetes, defined using either impaired fasting glucose or impaired glucose tolerance regardless of criteria. Lifestyle interventions were classified as dietary, exercise, and/or behavioural, and did not include herbal remedies or nutraceuticals (such as n-3 fatty acid supplements) or interventions of single dietary aspects (i.e. GI index, fibre, meal replacements, etc.). Interventions that were exclusively focused on changing behaviours of health professionals or surgical interventions were also excluded from this analysis. Comparison group(s) could include either usual care or a similar intervention at differing intensity (ie. physical activity or diet program alone). Primary study outcomes were incident diabetes, mortality, cardiovascular disease, diabetes complications, and quality of life. Secondary outcomes included glycemic control (either fasting glucose or HbA1c), hypertension, and/or blood pressure. To be included, articles had to report on at least one primary or secondary outcome, though these outcomes were not the subject of our analysis. All eligible articles were subject to thematic content analysis to explore the research questions and identified through clinical trial registries to enhance our analysis. Specifically, we collected registered participant inclusion criteria and outcomes (primary and secondary) related to body weight and compared to what was published in our included articles.

### Analysis

All articles were uploaded into NVivo 10 (QSR International) for analysis and analyzed using the framework method as described by Gale and colleagues [19] as well as holistic coding, which applies a single code to each large unit of data to capture overall content as well as possible categories [20]. All references to “obesity” and “weight” were identified, tabulated, and assigned an in vivo code, a form of coding that labels units of data according to the literal language present in the data source [20]. In vivo codes were subsequently subject to second-cycle concept coding in which the literal codes were assigned to categories based on their underlying meaning [20]. Lastly, patterns among the codes were identified inductively among conceptual categories. Similar, inter-related categories were then collapsed into themes. Rigor was enhanced through ongoing peer debriefing, investigator triangulation, and literature review [21]. Additional themes and conceptual categories, other than what is presented in the present article are forthcoming [22].

## Results

### Search results

**Fig. 1:**
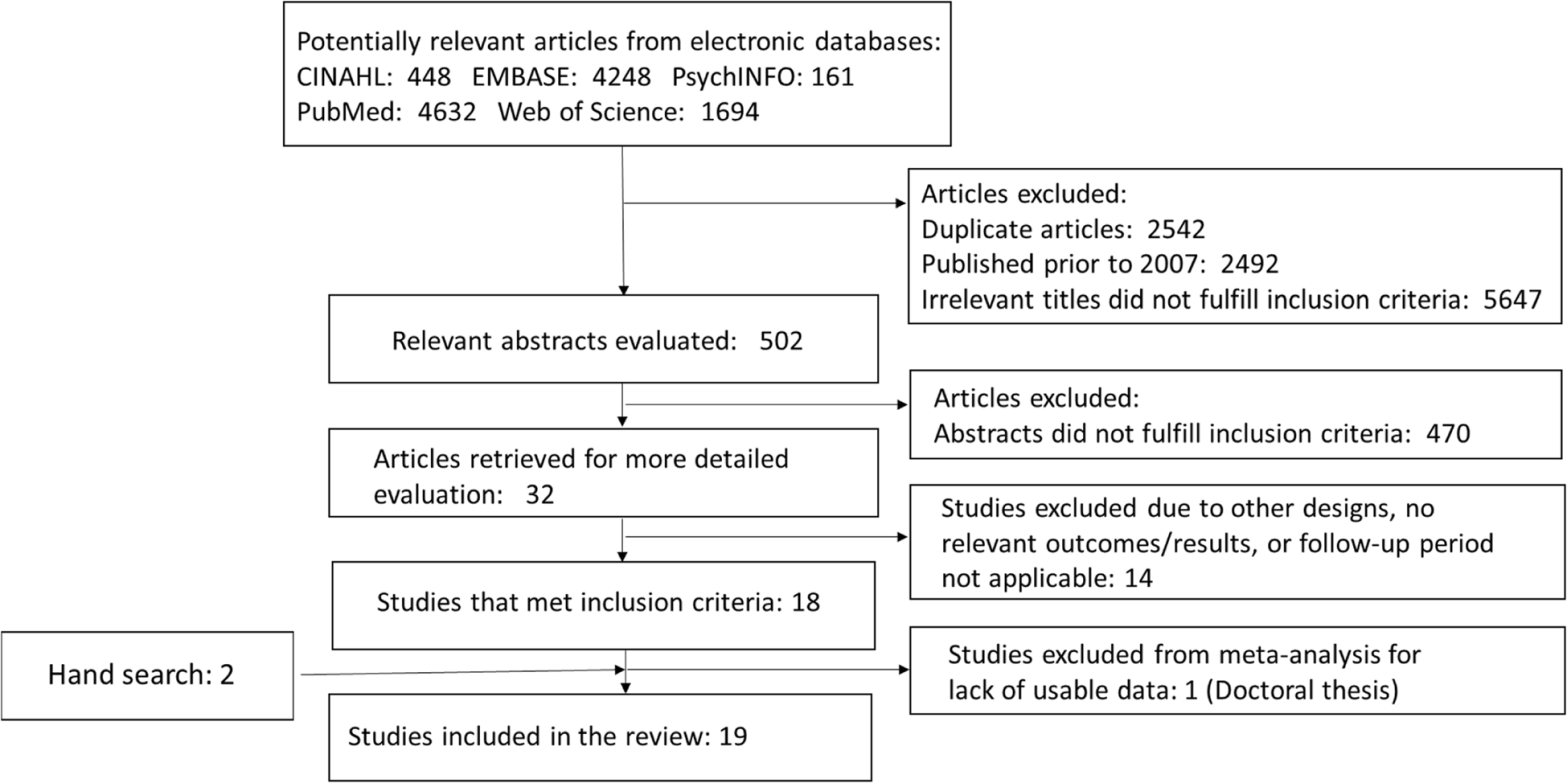
Systematic search results

The search retrieved 11,183 references. NR, AM and JB screened all titles, excluding duplicate articles (*n* = 2542) and those published outside of the selection window (*n* = 2492). Following this, abstracts deemed appropriate were evaluated by AM and NR, of which 32 full articles were retrieved for further, more detailed evaluation. The final selection excluded articles using different study designs, those with a follow-up period < 12 months, as well as those with ineligible outcomes, as described in our methods (*n* = 13). Additionally, one study was excluded due to lack of usable data because it was a thesis; it was considered too unwieldy to analyze qualitatively (Fig. 1). In total, 19 papers fulfilled the search criteria and were included in our qualitative analysis (Appendix 2). Of the 19 studies, half included data collected in the USA, with the remaining studies gathering data from Japan, Finland, Australia, Denmark, and Sweden. Additionally, five studies reported results from the Action for Health in Diabetes (Look AHEAD) trial (Alonso et al., 2015; Jakicic et al., 2013; Look AHEAD, 2010; Look AHEAD, 2013; Zhang et al., 2016), which was an RCT originally designed to determine whether intentional weight loss and increased physical activity would reduce cardiovascular morbidity and mortality in overweight individuals with type 2 diabetes undertaken in 16 American centres.

Studies included reported on various primary and secondary study outcomes, including incident diabetes (*n* = 6), mortality (*n* = 2), cardiovascular disease (*n* = 4), diabetes complications (*n* = 2), quality of life (*n* = 2), glycemic control (*n* = 18), and hypertension and/or blood pressure (*n* = 12). Despite intentional exclusion of the terms “weight loss” and “obesity” in the initial search, all studies observed change in body weight as an outcome to assess effectiveness of interventions. Fourteen of the articles had their trials registered through clinicaltrials.gov (including multiple articles from one trial). An additional two trials were found to be registered at UMIN-CTR Clinical Trial. Of the 16 articles with registered trials, nine did not register weight loss, obesity, body weight, or BMI as a primary or secondary outcome. The overarching theme that emerged in our qualitative analysis was *spin reporting* (Table 1).

**Table 1:** Summary of qualitative analysis, including examples of analytical categories identified

Examples	Description	Analytical Categories
“Cox-regression analysis of pooled (intervention and control group) data, showed that participants who achieved ≥5% weight loss at year one had 64% lower T2D incidence….” (Penn et al. 2013:5 and Fig. 4) “Results of these analyses showed that a greater increase in fitness was associated with greater weight loss with DSE [Diabetes support and Education] and ILI [Intensive Lifestyle Intervention] combined…” (Jakicic et al. 2013: 1301 and Fig. 3) “There was a significant reduction in waist circumference at 3 months (− 2.5 cm; 95% CI -3.8 to − 1.3) and 1 year (− 2.8 cm;95% CI -4.4 to − 1.1) in the intervention group” (Juul et al. 2016;116) “With LTPA treated as a continuous variable, there was a significant association (*p* = 0.02) between change in LTPA and change in weight after adjustment for baseline LTPA, clinic, treatment, clinic*treatment, and baseline weight. This association remained significant when data were analyzed separately for ILI (*p* = 0.02) but not for DSE. With change in LTPA treated as a continuous variable and with adjustment for selected covariates, there was no significant association observed between HbA1c and LTPA”. (Jakicic et al. 2013;1301) “The initially large effects in HRQOL that diminish over time are consistent with the larger weight loss and greater compliance with the intervention seen in the first year of Look AHEAD followed by weight regain and diminished compliance in later years” (Zhang et al. 2016; 862) “Another possibility is that a sustained weight loss of more than that achieved in the intervention group may be required to reduce the risk of cardiovascular disease” (Wing et al. 2013; 151) “These new findings advance our knowledge that community delivery of the DPP intervention in a lower-cost format by the YMCA can achieve meaningful weight loss among broader segments of the US population. This has important implications for ongoing diabetes prevention” (Ackermann 2015; 2333) “Weight loss is assumed to be the predominant factor for preventing type 2 diabetes in high risk groups” (Juul et al. 2016; 118) “Analysis showed the preventive effect of 5% weight loss, especially if maintained long term, which has utility for intervention monitoring” (Penn et al. 2013) “Most importantly, we found that this relatively modest intervention could produce beneficial effects on the incidence of type 2 diabetes during a 3-year period. The halving (51%) of the relative risk for overall subjects through this intervention is not negligible, even though it did not reach a statistically significant level”. (Sakane et al. 2011; 6)	Pooling of intervention and control group data to test for association between weight loss and outcomes Within group differences reported before or instead of between group differences Prioritizing positive secondary outcomes over non-significant primary outcomes Significant change in secondary outcomes presented as certain to produce reduction in diabetes incidence or complications Weight loss presented as an objective measure of lifestyle adherence, particularly in the context of lifestyle behavioural data being unreliable Varying BMI cut-points used for inclusion criteria in addition to measures of glucose metabolism Weight loss, regardless of intervention group or the inclusion of primary outcomes, was presented as certain to reduce risk of diabetes and its complications	Pooling data Within group difference Promote positive secondary outcome Downplay negative primary outcome Order of presentation Alignment of published outcomes with trial registry Omission of weight loss as outcome in registry Caloric restriction Dietary data not available/unreliable BMI as inclusion criteria Weight as adherence Weight loss as equivalent to diabetes risk reduction

### Pooling data

Spin reporting was consistently observed in all articles to varying degrees and in many forms. A common form of spin deployed in the reviewed articles was the pooling of intervention and control group data to test for associations of weight loss with other outcomes. “By pooling data from intervention and control groups for the analysis of weight loss and T2D incidence, we sought to evaluate the utility of sustained weight loss as an intermediate health outcome” (Penn et al. 2013; 5). Pooled data was also presented in various figures and tables, particularly with respect to weight loss. The graphical and tabular depiction of pooled weight loss data helped to cement the importance placed on weight loss in the publications - despite *lifestyle change* being the intervention of all included RCTs. This approach effectively eliminates the strength of an RCT design – and transforms interventional data into observational data.

### Within group differences

Authors also reported within group differences rather than, or prior to, between-group differences - despite this being counter to RCT design; for example, Christian et al. (2008;144), states, “Although 59% of the intervention patients experienced reductions in HbA1c level, this was not significantly different”. Non-significant primary outcomes were often minimized by emphasizing the direction/magnitude of change in the lifestyle group (despite non-significance) and/or prioritizing secondary positive outcomes. For instance, Juul and colleagues (2016; 118) write, “A brief theory-based health promotion intervention provided in the community indicated positive effect on weight, waist circumference and systolic blood pressure over one year among Danish adults at high risk of type 2 diabetes. However, there were no statistically significant differences in the primary outcomes of the DPS behavioural goals at one year”. Weight loss was also commonly reported first in the series of results, giving the impression of more importance of these results, compared to even primary outcomes.

### Weight as multiple measures

Body weight was not consistently described as one type of measure throughout the studies. Body weight measures [Body Mass Index (BMI) or weight] were discussed as inclusion criteria, a measure of adherence to lifestyle intervention, an intermediate outcome, and/or as a study outcome. These inconsistencies are in alignment with the discrepancies described above concerning registered trial outcomes.

BMI was included as inclusion criteria in all but five studies, with varying BMI cut-off points between studies but generally above 24 kg/m2 (i.e. overweight). Body weight was presented as a key characteristic in identifying individuals at-risk of developing diabetes and complications, even when diabetes, fasting glucose, or oral glucose tolerance testing were also used as inclusion criteria.

Authors justified pooling intervention and control groups and analyzing these data to test for relationships between weight loss and outcomes by arguing that doing so revealed the true intervention effect. In this way, weight loss was insinuated to signal dietary or exercise changes in line with the intended intervention, regardless of whether weight loss occurred in the intervention or control group. This notion was further reiterated when dietary data were deemed unreliable by some authors. In this context, weight loss was presented as an objective measure of intervention adherence. “Weight loss is attractive for monitoring compliance with interventions in routinely provided services, because it can be easily and objectively measured” (Penn et al., 2013; 8).

### Weight and conclusions

Weight loss was also presented as certain to lead to diabetes prevention, irrespective of whether the studies included diabetes incidence as an outcome (Ackerman et al., 2015; Marrero et al., 2015; Nilsen et al., 2011). Ackerman et al. (2015;2333) pronounce that “modest weight losses translate into fewer cardiovascular events, lower health care consumption, and better quality of life for most community-dwelling adults with prediabetes”. Authors discussed and concluded with varying degrees of certainty that significant weight losses observed in lifestyle intervention groups demonstrates the intervention’s capacity to prevent diabetes. “These data suggest that Weight Watchers, a widely available, empirically validated weight-management program, could offer a potential tool to significantly expand access to diabetes prevention programs in community settings and produce weight-loss levels that translate into considerable reductions in diabetes risk” (Marrero et al. 2016; 955). The misalignment of reported results with conclusions was a common type of spin, particularly with respect to the leap from weight loss to diabetes prevention.

## Discussion

In summary, despite the exclusion of weight-related search terms, obesity and weight loss figured prominently in the rationale, results, and interpretation of the articles. It was unclear whether weight loss was the intervention itself, a measure of adherence, or a study outcome. How studies were reported in trial registries only contributed to this confusion. Spin reporting was manifested in the focus on weight loss placed throughout the articles (contrary to design and registry outcomes) including in reporting of results; how the pooling of results produced the observational analysis of weight loss and various outcomes; and the reporting of within group differences prior to, or instead of, between group differences.

The incongruence of registered versus published outcomes is similar to RCTs in other fields [13–15]. Researchers, reviewers, and editors must take greater care to ensure alignment between registration and publication. Registration of RCTs was undertaken primarily to minimize publication bias, and selective reporting of outcomes, among other reasons. However, without appropriate verification, the purpose of registration may not be met. This misalignment may be partially explained by researchers’ lack of agreement on whether weight loss is in fact an (intermediate) outcome, a measure of intervention adherence, or the intervention itself. Without this explicit distinction, it is difficult to interpret the data and more importantly, the implications of lifestyle interventions for diabetes prevention.

Spin reporting, including all types reported here, have also been previously described among RCTs in other fields [16, 23–25]. Thus this finding is not specific to obesity or diabetes research but perhaps partially characteristic of RCT reporting. Spin reporting, in other fields, has also been associated with industry funding [26]. Though we did not investigate any role for industry funding in our analysis, it should be mentioned that many industries benefit from the personal responsibility framing of obesity and, by extension, type 2 diabetes.

RCTs require substantial investment of time and resources, particularly when disease incidence is the primary outcome and length of follow-up is long. This context, combined with pressure to publish and potential hardship related to publishing non-significant findings, may contribute to spin reporting in general. Notably, a recent meta-analysis of RCTs, using similar inclusion criteria as the present study, indicated significant publication bias in this area such that approximately 9 studies with null effects were estimated to be missing [27]. Unique to the present study and our related critical social science analysis [22] is the qualitative investigation of language and the potential role that social, and often moralistic, presentations of body weight may play in spin reporting and how results are presented, unlike many other health-related conditions. In turn, spin reporting in RCTs has been demonstrated to influence readers’ interpretation of study results [28], which may be especially problematic given the prominence of the “gold standard” RCT design. Boutron and Ravaud [29] have previously discussed potential strategies for mitigating spin through changes to the academic reward system.

Five studies did not include BMI as inclusion criteria. This, combined with the emphasis on weight loss in most studies, is problematic as it leaves little room in the “gold standard” research base for producing valuable knowledge on weight changes as a symptom of diabetes, and individuals with diabetes, impaired glucose tolerance, or impaired fasting glucose who have a “healthy” BMI. Ethnic minorities represent a disproportionate number of these cases [30]. Some studies attempted to account for this by including lowered BMI cut-points for inclusion criteria of participants of Asian descent (ex. Marrero et al. 2016). Several authors acknowledged the important role ethnicity plays in the relationship between body weight and diabetes, and the limited research in this area. Notably, three of the studies without BMI inclusion criteria included exclusively Japanese participants. The overrepresentation of weight loss trials for diabetes prevention was framed as important rationale by Sone et al. (2010) for their trial with Japanese participants without a specific focus on weight loss or inclusion of only overweight or obese participants. Relevantly, the trial by Sakane and colleagues (2011), which included Japanese participants of any BMI found that lifestyle intervention significantly reduced diabetes incidence only among the sample with BMI > 22.5. Taken together, the existing focus of lifestyle interventions aimed at weight loss for type 2 diabetes among overweight/obese participants may represent a form of inequity towards ethnic minorities by way of analytic omission. Importantly, India and China have the highest number of people living with diabetes among all countries globally [31].

Intermediate or surrogate outcomes are much more common among RCTs as compared to observational studies due to the long follow-up period required in RCTs to obtain sufficient statistical power for analysis of clinical end points. Regardless of these limitations, surrogate outcomes must be validated. In this way, weight loss must have been shown to predict clinically important outcomes, such as diabetes incidence [32]. However, the relationship between weight loss and diabetes incidence remains disputable; despite early confirmatory results in the Diabetes Prevention Program, diabetes incidence was not significantly different between lifestyle and control groups at 10-year follow-up, regardless of greater initial weight loss among the lifestyle group [3]. Furthermore, several observational studies support an inverse relationship between BMI and mortality among individuals with diabetes [33, 34], and weight loss among those with diabetes is not necessarily beneficial [35, 36]. Importantly, weight loss is rarely sustainable due to involuntary, homeostatic pressures [37, 38]. This large and growing body of research disputes the validity of weight loss as necessarily a surrogate measure for diabetes prevention.

The practical implications of the over-reliance on weight loss as a surrogate outcome for diabetes prevention needs to be considered as well, particularly given that its validity is questionable. Firstly, the role of weight loss in diabetes prevention may have particular incongruencies with patient-oriented care; and secondly, weight bias in health care may be an added barrier to diabetes management [39] and cardiovascular care [40]. Velentgas and colleagues [41] report that patients often are less concerned with intermediate pathways without clear links to clinical impact. This may be further magnified by how researchers make assumptions concerning participants’ adherence/compliance based on weight loss measures [22], which eliminates a) the truly democratic shared decision model espoused in patient-centred practice [42, 43]; and b) may contribute to patients fearing clinicians’ assumptions regarding lifestyles and ultimately, treatment avoidance [40].

Researchers must more carefully assess how they discuss participants in terms of compliance, particularly with respect to highly stigmatized conditions such as obesity and diabetes. Researchers must reflect on their own views and biases in data interpretation and how this may translate into clinically important assessments of symptoms, functioning, and quality of life [44]. Ultimately, patients and practitioners want trusting, mutually-respectful relationships in which they feel heard, and physicians and patients agree that empathy and respect are essential when addressing weight in health care settings [45]. Essential to this patient-centred agenda is an evidence-base that does not over-emphasize weight, blame patients for intervention failures, simplify weight issues to a matter of adherence, or reinforce a moralistic imperative (good vs. bad patients) concerning patient adherence [46].

This study is subject to limitations. First, articles published in a language other than English were excluded, thus constituting our own reporting bias. Second, our thematic analysis was limited to the ways in which body weight may have introduced bias in the development and reporting of RCTs; no other forms of bias were examined. Third, our search protocol is not registered.

## Conclusions

This thematic analysis of RCTs to prevent diabetes found evidence to reinforce critiques raised by others that obesity research has come to be characterized by the reiteration of a series of infrequently challenged myths, presumptions, and suppositions [8, 9]. Our study suggests that even in valuable, carefully designed RCTs, there is an entrenchment of certain biases regarding obesity, weight loss, and diabetes prevention. These biases influence the analyses, interpretation and depiction of results concerning weight, diet, and exercise in diabetes interventions, which in turn may reinforce stereotypes that an individuals’ body weight is a result of their diet and exercise, and lack of weight loss indicates lack of compliance with an intervention. Ultimately, facile interpretations carry political implications in terms of research funding,^8^ and this affects the lives and health of the general public, those at risk for diabetes, and those already living with diabetes. While registration of full trial protocols with a priori information on secondary analyses is necessary to begin to address reporting bias, it would not be sufficient. A patient-oriented approach to research and clinical practice that is based on respect and empathy is an important step in shaping future research in diabetes prevention and management.

## Appendix 1

### Search terms for study selection

(“Diabetes Mellitus Type 2”[Mesh] OR maturity-onset diabetes of the young[Title/Abstract] OR MODY[Title/Abstract] OR dm2[Title/Abstract] OR niddm[Title/Abstract] OR iidm[Title/Abstract] OR non insulin depend*[Title/Abstract] OR noninsulin depend*[Title/Abstract] OR type 2 diab*[Title/Abstract] OR type II diab*[Title/Abstract] OR ketosis resistant diabetes[Title/Abstract] OR adult onset diabet*[Title/Abstract] OR late onset diabet*[Title/Abstract] OR maturity onset diabet*[Title/Abstract] OR slow onset diabet*[Title/Abstract] OR stable onset diabet*[Title/Abstract] OR stability onset diabet*[Title/Abstract] OR plurimetabolic syndrome[Title/Abstract]) AND (“Exercise”[Mesh] OR “Physical Education and Training”[Mesh] OR “Physical Fitness”[Mesh] OR “Life Style”[Mesh] OR “Health Education”[Mesh] OR “Health Behavior”[Mesh] OR “Health Promotion”[Mesh] OR “Sports”[Mesh] OR “Physical Exertion”[Mesh] OR “Exercise Therapy”[Mesh] OR “Nutrition Therapy”[Mesh] OR “Diet Therapy”[Mesh] OR “Feeding Behavior”[Mesh] OR “Running”[Mesh] OR “DietDiabetic”[Mesh] OR “Jogging”[Mesh] OR “Swimming”[Mesh] OR “Walking”[Mesh] OR “Bicycling”[Mesh] OR exercise[Title/Abstract] OR exercising[Title/Abstract] OR exertion*[Title/Abstract] OR sport[Title/Abstract] OR sports[Title/Abstract] OR walking[Title/Abstract] OR jogging[Title/Abstract] OR swimming[Title/Abstract] OR strength train*[Title/Abstract] OR resistance train*[Title/Abstract] OR aerobic train*[Title/Abstract] OR physical education*[Title/Abstract] OR physical fitness[Title/Abstract] OR nutrition[Title/Abstract] OR nutritional [Title/Abstract] OR life style[Title/Abstract] OR lifestyle[Title/Abstract] OR health behav*[Title/Abstract] OR health educ*[Title/Abstract] OR health promot*[Title/Abstract] OR physical activit*[Title/Abstract] OR bicycling[Title/Abstract] OR weight lift*[Title/Abstract] OR running[Title/Abstract] OR gymnastic*[Title/Abstract] OR dance[Title/Abstract] OR dancing[Title/Abstract]OR diet[Title/Abstract]) NOT (“Dermatomyositis”[Mesh] OR “Myotonic Dystrophy”[Mesh] OR “Diabetes Insipidus”[Mesh] OR “DiabetesGestational”[Mesh]) AND (“humans”[MeSH Terms] AND English[lang] AND “adult”[MeSH Terms]) AND (Clinical Trial[ptyp] OR Randomized Controlled Trial[ptyp] OR randomized[Title/Abstract] OR placebo[Title/Abstract] OR randomly[Title/Abstract] OR trial[Title])

## Appendix 2

**Table 2:** List of publications included in the review

Ackermann RT, Liss DT, Finch EA, Schmidt KK, Hays LM, Marrero DG, Saha C. A randomized comparative effectiveness trial for preventing type 2 diabetes. Am J Public Health. 2015;105(11):2328–2334.	
Alonso A, Bahnson JL, Gaussoin SA, Bertoni AG, Johnson KC, Lewis CE, Vetter M, Mantzoros CS, Jeffery RW, Soliman EZ, Look AHEAD Research Group. Effect of an intensive lifestyle intervention on atrial fibrillation risk in individuals with type 2 diabetes: the Look AHEAD randomized trial. Am Heart J. 2015;170(4):770–777.	
Christian JG, Bessesen DH, Byers TE, Christian KK, Goldstein MG, Bock BC. Clinic-based support to help overweight patients with type 2 diabetes increase physical activity and lose weight. Arch Intern Med. 2008;168(2):141–146.	
Jakicic JM, Egan CM, Fabricatore AN, Gaussoin SA, Glasser SP, Hesson LA, Knowler WC, Lang W, Regensteiner JG, Ribisl PM, Ryan DH. Four-Year Change in Cardiorespiratory Fitness and Influence on Glycemic Control in Adults With Type 2 Diabetes in a Randomized Trial. Diabetes Care. 2013;36(5):1297–1303.	
Juul L, Andersen VJ, Arnoldsen J, Maindal HT. Effectiveness of a brief theory-based health promotion intervention among adults at high risk of type 2 diabetes: One-year results from a randomized trial in a community setting. Prim Care Diabetes. 2016;10(2):111–120.	
Lindahl B, Nilssön TK, Borch-Johnsen K, Røder ME, Söderberg S, Widman L, Johnson O, Hallmans G, Jansson JH. A randomized lifestyle intervention with 5-year follow-up in subjects with impaired glucose tolerance: pronounced short-term impact but long-term adherence problems. Scand J Soc Med. 2009;37(4):434–442.	
Look AHEAD Research Group. Long term effects of a lifestyle intervention on weight and cardiovascular risk factors in individuals with type 2 diabetes: four year results of the Look AHEAD trial. Arch Internal Med. 2010;170(17):1566.	
Look AHEAD Research Group. Cardiovascular effects of intensive lifestyle intervention in type 2 diabetes. N Engl J Med. 2013;369:145–154.	
Marrero DG, Palmer KN, Phillips EO, Miller-Kovach K, Foster GD, Saha CK. Comparison of commercial and self-initiated weight loss programs in people with prediabetes: a randomized control trial. Am J Public Health. 2016;106(5).	
Moncrieft AE, Llabre MM, McCalla JR, Gutt M, Mendez AJ, Gellman MD, Goldberg RB, Schneiderman N. Effects of a Multicomponent Life-Style Intervention on Weight, Glycemic Control, Depressive Symptoms, and Renal Function in Low-Income, Minority Patients With Type 2 Diabetes: Results of the Community Approach to Lifestyle Modification for Diabetes Randomized Controlled Trial. Psychosom Med. 2016;*78*(7):851.	
Nilsen V, Bakke PS, Gallefoss F. Effects of lifestyle intervention in persons at risk for type 2 diabetes mellitus-results from a randomized, controlled trial. BMC Public Health. 2011;11(1):893.	
Penn L, White M, Lindström J, den Boer AT, Blaak E, Eriksson JG, Feskens E, Ilanne-Parikka P, Keinänen-Kiukaanniemi SM, Walker M, Mathers JC. Importance of weight loss maintenance and risk prediction in the prevention of type 2 diabetes: analysis of European Diabetes Prevention Study RCT. PLoS One. 2013;8(2):e57143.	
Saito T, Watanabe M, Nishida J, Izumi T, Omura M, Takagi T, Fukunaga R, Bandai Y, Tajima N, Nakamura Y, Ito M., for the Zensharen Study for Prevention of Lifestyle Diseases Group. Lifestyle modification and prevention of type 2 diabetes in overweight Japanese with impaired fasting glucose levels: a randomized controlled trial. Arch Intern Med. 2011;171(15):1352–1360.	
Sakane N, Sato J, Tsushita K, Tsujii S, Kotani K, Tsuzaki K, Tominaga M, Kawazu S, Sato Y, Usui T, Kamae I, Yoshida T, Kiyohara Y, Sato S, Kuzuya H. Prevention of type 2 diabetes in primary healthcare setting: three-year results of lifestyle intervention in Japanese subjects with impaired glucose tolerance. BMC Public Health. 2011;11:40.	
Schultz MG, Hordern MD, Leano R, Coombes JS, Marwick TH, Sharman JE. Lifestyle change diminishes a hypertensive response to exercise in type 2 diabetes. Med Sci Sports Exerc. 2011;764–769.	
Shibayama T, Kobayashi K, Takano A, Kadowaki T, Kazuma K. Effectiveness of lifestyle counseling by certified expert nurse of Japan for non-insulin-treated diabetic outpatients: a 1-year randomized controlled trial. Diab Res Clin Pract. 2007;76(2):265–268.	
Sone H, Tanaka S, Iimuro S, Oida K, Yamasaki Y, Oikawa S, Ishibashi S, Katayama S, Yamashita H, Ito H, Yoshimura Y. Long-term lifestyle intervention lowers the incidence of stroke in Japanese patients with type 2 diabetes: a nationwide multicentre randomised controlled trial (the Japan Diabetes Complications Study). Diabetologia. 2010;53(3):419–428.	
Tuomilehto H, Peltonen M, Partinen M, Lavigne G, Eriksson JG, Herder C, Aunola S, Keinänen-Kiukaanniemi S, Ilanne-Parikka P, Uusitupa M, Tuomilehto J. Sleep duration, lifestyle intervention, and incidence of type 2 diabetes in impaired glucose tolerance. Diabetes Care. 2009;32(11):1965–1971.	
Zhang P, Hire D, Espeland MA, Knowler WC, Thomas S, Tsai AG, Glick HA. Impact of intensive lifestyle intervention on preference-based quality of life in type 2 diabetes: Results from the Look AHEAD trial. Obesity. 2016;24(4):856–864.	

## Abbreviations

BMI: Body mass index
RCT: Randomized controlled trial
